# Maternal weight gain adequacy is associated with newborn birth weight in gestational diabetes mellitus

**DOI:** 10.1186/1758-5996-7-S1-A81

**Published:** 2015-11-11

**Authors:** Livia Silveira Mastella, Leticia Schwerz Weinert, Vanessa Gnielka, Vânia Naomi Hirakata, Maria Lúcia Rocha Oppermann, Sandra Pinho Silveiro, Angela de Azevedo Jacob Reichelt

**Affiliations:** 1 Particular clinic, Porto Alegre, Brazil

## Background

Gestational diabetes mellitus (GDM) is diagnosed in up to 18% of Brazilian women and is commonly related to pre-gestational obesity; superimposed weight gain during pregnancy may adversely contribute to maternal and fetal outcomes.

## Objective

To evaluate weight gain patterns in women with GDM and its relation to newborn birth weight.

## Materials and methods

Cohort study including 362 GDM women classified according to their pre-gestational body mass index (BMI). Gestational weight gain (GWG) was considered insufficient, adequate or excessive based on the Institute of Medicine 2009 recommendations for each pre-pregnancy BMI class. Newborns were classified as large (LGA), appropriate (AGA) or small for gestational age (SGA) according to the Alexander curve.

## Results

Total GWG was 10.1±7.5 kg (range: -8.0 to 36.3 kg). Appropriate weight gain occurred in 25% of women, excessive in 37.5% and insufficient, in 37.5%. Adverse maternal outcomes (cesarean section, hypertension and postpartum dysglycemia) were similar among groups, as were prematurity rates. Obese women with excessive GWG had higher rates of LGA when compared to the other groups (28% vs 8% (insufficient) vs 6% (adequate), p=0.003). SGA rates were higher in women with normal BMI and insufficient GWG compared with the appropriate weight gain group (24% vs 0%; p=0.002).

## Conclusion

In women with GDM, excessive GWG, especially in obese women, was associated with increased risk of LGA. Insufficient weight gain was associated with increased SGA in women with normal BMI. Only 25% of women gained weight as recommended by Institute of Medicine 2009.

